# Is There an Association between Physical Activity and Sleep in Community-Dwelling Persons with Dementia: An Exploratory Study Using Self-Reported Measures?

**DOI:** 10.3390/healthcare7010006

**Published:** 2019-01-05

**Authors:** Emma Bartfay, Paige Stewart, Wally Bartfay, Efrosini Papaconstantinou

**Affiliations:** Faculty of Health Sciences, University of Ontario Institute of Technology, Oshawa, ON L1H 7K4, Canada; paige.stewart@uoit.net (P.S.); wally.bartfay@uoit.ca (W.B.); efrosini.papaconstantinou@uoit.ca (E.P.)

**Keywords:** dementia, Alzheimer’s disease, physical activity, non-pharmacological interventions, sleep quality, sleep quantity, quality-of-life, caregivers, community-dwelling

## Abstract

Sleep disturbances are common in persons with dementia (PWD). While pharmacotherapy is widely used, non-pharmacological interventions are beginning to surface as first-line management strategies. This study sought to investigate if physical activity was associated with more favourable sleep patterns in PWD, and to compare the sleep quantity and quality between active and inactive PWD. We conducted an exploratory study to tackle these research questions. Self-reported telephone questionnaires were administered to 40 caregivers of PWD, who answered questions as proxies on behalf of their care recipient. Just over half (55%) of our participants met the criteria for being active. Walking was the most popular form of physical activity for both active and inactive PWD. Active PWD also preferred exercise classes and gardening, whereas inactive PWD favoured chair exercises. Compared to their inactive counterparts, active PWD were more likely to experience appropriate sleep quantity (*p* = 0.00). The active group also reported significantly better overall sleep quality (*p* = 0.003). Together, our findings suggest that physical activity may be associated with improved sleep in PWD. Future studies are warranted to investigate whether physical activity can be promoted as a safe and effective means to improve quality-of-life in this population.

## 1. Introduction

Sleep is an essential component for the physical and mental well-being of an individual. Despite the overwhelming evidence surrounding the importance of sleep, the prevalence of sleep disturbances remains high in the older adult population [1,2]. Typically, sleep disturbances interfere with the normal sleep process leading to inadequate amounts of sleep and/or declined sleep quality. These changes can impair social functioning and increase an individual’s risk of cognitive errors and physical accidents [3,4].

Age-related changes place older adults at an increased risk of experiencing sleep problems, including those living with dementia. To make matters worse, the neurodegenerative aspects of dementia are said to contribute and compound the normal changes in sleep patterns that typically occur with aging [5]. As a result, the risk of lower quality-of-life due to sleep disturbances is much greater in persons with dementia (PWD). Far from being solely a medical concern, dementia has the ability to affect all areas of an individual’s and their family’s lives [6], and treatment options often focus on maintaining the quality-of-life for PWD and their caregivers.

Chronic sleep disturbances are common and they represent a debilitating characteristic of dementia [7,8]. These disturbances are frequently manifested as difficulty sleeping at night, excessive daytime sleepiness, and an increase in sun-downing behaviour. Specifically, individuals in the late stages of dementia are said to spend as much as 40 percent of their time in bed at night awake, and sun-downing occurs in approximately 12–25% of individuals with Alzheimer’s disease [9]. These behaviours can be very disruptive and harmful, and often require a combination of pharmacological, behavioural, and/or environmental interventions.

The pathophysiological relationship between dementia and sleep is not completely understood. Previous studies have identified excessive damage that results from dementia in the neuronal pathways located in the suprachiasmatic nucleus (SCN) as a potential cause [5,10]. As suggested by Van Erum and colleagues [10], the changes in the SCN during the course of dementia may have affected the circadian rhythm, where these circadian disturbances form the root of sleep-wake problems. As a result, the impairment due to dementia greatly impacts an individual’s ability to maintain a proper sleep–wake cycle. Another contributing factor may be the markedly decreased melatonin levels in PWD. Evidence suggests that melatonin levels in PWD are 80% less than the levels in age-matched individuals without dementia [9]. These changes may exacerbate the severity of sleep problems that typically occur with aging, leading to a decrease in sleep quality and quantity for PWD and their primary caregivers.

Livingston and colleagues [11] estimated that 40% of PWD experienced disturbed sleep, and we are yet to find a safe and effective form of treatment for this problem. Pharmacotherapy for the management of sleep problems in PWD is in the form of anti-depressants, anti-psychotics, or sleeping pills. Although it is the most common intervention currently used, the evidence regarding its effectiveness to improve sleep is inconclusive [12,13]. Furthermore, these drugs can have serious side-effects such as an increased risk of falls or disorientation. As a result of these concerns, non-pharmacological interventions (NPIs) are beginning to gain recognition as a first-line approach to manage sleep problems and improve the quality of sleep. The NPIs most frequently cited in the literature include light therapy, sleep hygiene, and physical activity (PA) [5]. The latter intervention is the subject of this investigation.

Although sparse, existing research has suggested positive results for the use of PA as an intervention to improve sleep in PWD. Nevertheless, the lack of homogeneity in these studies makes it difficult to determine what mechanism of PA is effective in improving sleep. Specifically, studies investigating this relationship often used mixed inclusion criteria, implemented various methods of PA, and utilized different outcome measures [14]. Furthermore, the PA component in the majority of these studies was often used in combination with other treatments such as sleep hygiene therapy, increased daytime bright-light exposure, social activities, and reducing environmental stimulants that may disrupt night-time sleep [15,16,17]. As a result, it was difficult to tease out the benefits of PA alone.

### Study Aims and Hypotheses

The main objective of this study was to explore the relationship between moderate PA and sleep in community-dwelling PWD. We were particularly interested in the types and levels of engagement in PA in this population. Two hypotheses were considered in this study: (i) We hypothesized that PWD who engaged in moderate PA had more appropriate quantity of sleep than their non-active counterparts, and (ii) we hypothesized that PWD who engaged in moderate PA had better quality sleep than their non-active counterparts.

## 2. Materials and Methods

### 2.1. Study Design

We conducted an exploratory study utilizing telephone interviews as our primary method of data collection. Although information was sought on PWD, their primary informal caregivers were recruited as proxy informants for the study. Location for recruitment included eight caregiver support groups and three public education sessions organized by the Alzheimer’s Society of Durham Region in Canada.

### 2.2. Study Procedure and Instruments

Prior to the conduction of the study, the research team had received institutional ethics approval (REB File #: 15-060) as well as the permission to undertake the study by the Alzheimer’s Society of Durham Region. All participants were recruited in-person. At the end of each caregiver support group meeting, attendees were introduced to the study background and procedures. Letters of information with attached consent forms were distributed. At the public education events, participants were introduced to the study at the beginning of the session, and were told where they could sign up once the session had ended. Individuals who were interested in the study were asked to complete a form that included a statement of consent and their contact information. Participants were then contacted at the time they indicated, in order to gain assent from the PWD, if they were capable, and to complete the survey. For our study, we defined primary caregiver as an individual who took principal responsibility for the care of a family member who had a diagnosis of dementia. A formal diagnosis of dementia by a health care provider was required, and the PWD must reside in the community and not in a long-term care facility.

During the telephone interview, the PWD was invited to participate alongside the caregiver if they desired, while the caregiver completed the questionnaire verbally. The entire interview lasted for approximately 10 min, which included the assent process for the PWD, completing the questionnaire, and debriefing study information. The questionnaire inquired about the demographics, PA engagement, and information about the sleep characteristics of the PWD. Sleep was evaluated using a modified version of the Medical Outcomes Study (MOS-SS). The MOS-SS is a 12-item questionnaire that has consistently demonstrated validity and reliability for assessing the impact of disease on sleep [18]. In particular, sleep quality was determined by latency until sleep onset, night-time awakenings and daytime sleepiness, whereas sleep quantity was measured as the length of time that the PWD sleeps on average per night. The National Sleep Foundation recommends 7–8 h of sleep, but considers 6 or 9 h acceptable. Anything outside of that range is deemed severe sleep restriction [19]. These values were used to determine if an individual was experiencing poor sleep quantity or if they were at an acceptable/optimal level of sleep.

PA level was measured as an individual’s total leisure-time physical activity energy expenditure (LTPAEE), expressed as kilocalories per kilogram of body weight per day (kkd), using this formula,

LTPAEE (kkd) = Σ [(*N_i_* × *D_i_* × MET_i_)/7]

where *i* indicates each activity being considered. *N_i_* and *D_i_* represent the number of times in a week and the duration for the *i*th activity. Adopted from the Canadian Fitness and Lifestyle Research Institute, each activity has an assigned metabolic expenditure of task (MET) value, which represents the metabolic energy cost of an activity [20]. After calculating the value for LTPAEE, PWD were classified as active if they engaged in ≥1.5 kkd of moderate PA. This cut-off point is equivalent to walking for 30 min per day, or being moderately active [21].

### 2.3. Data Analysis

Statistical Package for Social Science version 22 (SPSS Inc., Chicago, IL, USA) was employed for data analysis. Descriptive statistics were performed to summarize and describe the data. Univariable comparisons between and among groups included chi-square tests, *t*-tests and ANOVA.

## 3. Results

### 3.1. Basic Demographic Information of Participants

A total of 40 primary caregivers participated in the study, all of whom provided information on the PWD they cared for. The average age of our participants was 78.6 (SD = 7.0). Slightly over half of the PWD involved were male (*n* = 23, 57.5%). The age range by sex was 65–89 and 73–92 for the male and female PDW, respectively. One-third had an educational level beyond secondary school (*n* = 13, 32.5%). Most participants were married or in a common-law relationship (*n* = 32, 80.0%), and about half used home care services (*n* = 18, 45.0%). The most common diagnosis was Alzheimer’s disease (*n* = 17, 42.5%), followed by vascular dementia (*n* = 6, 15.0%). Half of our participants had their diagnosis made between 1 and 40 months ago (*n* = 19, 50.0%) and three participants had their diagnosis made 8–10 years ago. Prescription drugs for dementia were used in approximately three-quarters of the participants (*n* = 31, 77.5%). In terms of sleep aids, the majority of our participants did not use any prescription (*n* = 31, 77.5%) or over-the-counter (OTC) sleep medication (*n* = 37, 92.5%).

### 3.2. Comparison of Active and Inactive PWD

Using the formula described earlier, 18 PWD were classified as ‘inactive,’ whereas 22 PWD met the criteria to be classified as ‘active’ (45.0 vs. 55.0%). Inactive and active PWD were similar in all demographic characteristics we examined, including age, gender, type of and time since diagnosis, marital status, education level, home care service and medication use. Although none of the differences in these characteristics was statistically significant, there were some notable disparities. For example, the active group comprised of more male PWD (68.2 vs. 44.4%, *p* = 0.13) and were more likely to be married or in a common-law relationship (85.7 vs. 77.8%, *p* = 0.52) than the inactive group. The active group was also more likely to use at least one prescription drug for dementia (88.2 vs. 76.2%, *p* = 0.34), with the most common drugs being Aricept, Exelon, Reminyl, and Galantamine. Conversely, the active group was less likely to use OTC sleep medication (4.6 vs. 11.1%, *p* = 0.43) or prescription sleep medication (13.6 vs. 33.3%, *p* = 0.14). Although not statistically significant, the differences in sleep medication use suggest that individuals who were inactive may be more likely to rely on such aids than their active counterparts. These findings are summarized in Table 1.

### 3.3. Type, Frequency and Duration of Physical Activities Performed by PWD

In terms of the type of PA, walking was the most frequently cited activity performed by both active and inactive PWD. Almost two-thirds (62.5%, *n* = 25) of respondents indicated that their loved one did some form of walking, and the majority of whom walked every day (45.0%, *n* = 18). Furthermore, results revealed that 86.4% (*n* = 19) of active subjects used walking as a form of PA compared to only six (33.3%) inactive PWD. The mean frequency of walking for inactive PWD was 1.9 days per week (SD = 3.0), compared to 5.4 days per week (SD = 2.6) for active PWD. On average, inactive participants walked for less than 5 min per week (0.07 h ± 0.12), compared to almost one hour per week in active participants (0.96 h ± 1).

In addition to walking, exercise classes and gardening were popular forms of PA in the active group. Both exercise classes and gardening were used as activities for 27.3% (*n* = 6) of active PWD one or more times per week. The least popular activities for this group were golfing, skiing, home exercises, tennis, biking, and chair exercises (exercises that involved sitting or holding on to a chair such as sit-to-stands, seated marches, and seated shoulder press). Activity preference varied slightly for inactive PWD. Although walking and exercise classes were still the two most commonly used activities, chair exercise was also a popular form of PA. In fact, a greater percentage of inactive subjects participated in chair exercise than their active counterparts (16.7% vs. 2.5%). They also preferred home exercise more than the active PWD (5.6% vs. 0%). Activities that had no participation in the inactive group were gardening, weight training, golfing, skiing, tennis, and cycling. These results are summarized in Table 2.

### 3.4. Comparison of Sleep Quantity

Two aspects of sleep benefits were considered in this study: sleep quantity and sleep quality. For the former, we followed the National Sleep Foundation’s recommended hours of sleep per night for individuals over 65 [22]. A sleep duration of 6–9 h per night was classified as ‘appropriate.’ Conversely, sleep durations of fewer than 6 h or greater than 9 h were considered ‘inappropriate.’ Our results showed that 19 active PWD were given an appropriate sleep rating, compared to only two PWD in the inactive group. The difference was statistically significant (*p* = 0.00), suggesting that PA was significantly associated with more favourable sleep quantity.

Prescription sleep medication use was another variable found to negatively affect sleep quantity. Among those who did not take prescription sleep medication, 19 (90.5%) were in the appropriate sleep quantity group, compared to 12 (63.2%) who were in the inappropriate sleep group. The difference was statistically significant (*p* = 0.04). Interestingly, we also found that PWD who were not prescribed medication for dementia were more likely to experience optimal sleep quantity. Specifically, 30.0% of PWD (*n* = 6) who were not on dementia medication achieved appropriate quantity, compared to only one PWD (5.6%) who was deemed to be severely sleep restricted. This difference was also statistically significant (*p* = 0.05). Nevertheless, this observation maybe related to the severity of the disease, and therefore, should be interpreted with caution. No other variable we considered was significantly associated with sleep quantity. These variables included age (*p* = 0.75), gender (*p* = 0.55), education (*p* = 0.37), marital status (*p* = 0.73), use of home care services (*p* = 0.36), type of dementia (*p* = 0.44), use of OTC sleep medication (*p* = 0.49), and time since diagnosis (*p* = 0.84). The results are summarized in Table 3.

### 3.5. Comparison of Sleep Quality

Our second sleep aspect was related to sleep quality. The Medical Outcomes Study Sleep Scale (MOS-SS) scoring instrument was used to determine an individual’s sleep quality. The scoring guide assigns each subject a sleep quality score out of 100 based on their responses to the survey questions relating to various aspects of sleep quality. Once this value was obtained, participants were classified into one of three categories. These categories were modelled after Viala-Danten and colleagues’ study [23]. A score of 0–30 represented good sleep quality, a score of 30.1–60 indicated moderate sleep quality, and 60.1–100 was considered poor sleep quality. Following this categorization, there were 11 PWD who experienced good sleep quality, 18 who had moderate sleep quality, and 11 with poor sleep quality.

The results showed that there was a greater number of active subjects in the good and moderate sleep quality groups. Furthermore, we found only one inactive PWD experienced good sleep quality, and only two active PWD experienced poor sleep quality. In fact, 90.9% of PWD who received the highest sleep quality grouping were in the active group. The difference was statistically significant (*p* = 0.003). Our results also showed that marital status had a marginally significant effect on sleep quality (*p* = 0.06). We noted that all PWD who experienced poor sleep quality were either married or in a common-law relationship while no single PWD experienced poor sleep quality. However, these results must be interpreted with caution since the majority of study participants were in the married/common-law category. Additionally, a spouse who shared a bed with a PWD was more likely to notice any sleep disturbances than other caregivers, such as an adult child, who likely did not share the same bed.

No other variable was found to be associated with sleep quality. Nevertheless, prescription sleep medication once again merited a note of mention, even though it was not statistically significant (*p* = 0.10). While no PWD who took prescription sleep medication experienced the highest level of sleep quality, 11 PWD (35.5%) who did not take prescription sleep medication experienced good sleep quality. Other non-significant variables included age (*p* = 0.78), time since diagnosis (*p* = 0.48), use of medication for dementia (*p* = 0.97), OTC sleep medication use (*p* = 0.92), gender (*p* = 0.89), use of home care services (*p* = 0.37), education (*p* = 0.47), and type of dementia (*p* = 0.36). These findings are summarized in Table 4.

## 4. Discussion

Dementia represents a significant public health concern that will continue to grow as the population ages. The main purpose of this research was to examine whether there is an association between physical activity and sleep in community-dwelling PWD. We also identified the level of PA that these individuals participated in, and what types of PA they preferred. Although previous studies have explored this topic to some extent, they often focused on institutionalized PWD. Furthermore, these studies often examined the combined effect of different non-pharmacological interventions, making it difficult to tease out the effects that are attributable to PA alone.

Our findings strongly supported the current knowledge [15,24,25,26] that regular PA was associated with more favourable sleep characteristics. Being physically active has been defined as achieving an activity level that was equivalent to walking for 30 min per day [20]. We were pleasantly surprised to find that 55% of PWD in our study met these criteria. In fact, this figure was higher than that of the general Canadian population, where only 43% of Canadians age 65 or above reported to be moderately active [27].

None of the demographic characteristics we examined were significantly different between active and inactive participants. Some notable differences included gender where we found more active males and more inactive females. This finding was not unsupported by previous research, which found that males were generally more active in their leisure-time with respect to moderate-intensity activities. Gender-bias may have played a role here, especially when investigations used typically male-dominated activities rather than other domains such as household work [28,29]. It was also noted that although not statistically significant, a greater number of married PWD were active than inactive. Considering women commonly live longer than men and frequently out-live their partner, a lack of marital support may have contributed to the decreased activity level.

We also found that walking was the most popular form of PA in both the active and inactive groups. Our results showed 63.4% of active PWD and one-third of inactive PWD who engaged in walking did so every day, suggesting that waking may have become a habit of daily living for those who used it as a form of PA. As walking was shown to be an effective and popular way to obtain adequate amount of PA [30,31], interventions that target sedentary PWD to increase their walking frequency and duration may be beneficial. Our study further found that gardening and exercise class were also popular forms of PA among active PWD. As gardening has been proven to be an enjoyable activity that requires minimal caregiver supervision [32,33], it should be promoted to improve sleep in the dementia population. An interesting finding in our study was that chair exercises were performed more frequently among inactive PWD. This finding may reflect the fact that active subjects typically engaged in more strenuous activities, as chair exercises have been identified as ‘low-intensity’ [34]. Our findings, however, suggest it may be beneficial to develop interventions that increase the intensity of chair exercise since it appears to be popular among PWD who do not typically engage in more intense forms of PA.

The first study hypothesis stated that PWD who engaged in moderate PA had more appropriate quantity of sleep than their non-active counterparts. Our results showed that there were more active PWD who achieved an appropriate sleep rating than the inactive PWD and the difference was statistically significant. Nevertheless, current evidence on the relationship between PA and sleep quantity remains inconsistent. While some [32] clearly demonstrated such a relationship, others did not [15,16]. Still, McCurry and colleagues [16] showed that there were improvements over night-time awakenings and sleep efficiency, although no benefits of sleep quantity were observed.

We also found that sleep quantity was negatively associated with the use of prescription sleep medication. PWD who took sedative medication experienced significantly poorer sleep quantity than those who did not. Indeed, there is a growing body of evidence advising against the use of prescription sleep medication due to its ineffectiveness and potentially dangerous side effects. Although sedative medication may have shortened the sleep onset latency, there was a lack of empirical evidence indicating that it helped maintain sleep throughout the night and that it was effective for long-term use [5]. Furthermore, sedative medication has been reported to have a rapid development of tolerance and offered no benefit over behavioural techniques [35,36]. Thus, what minimal improvements it offers for sleep may diminish quickly. This may be one of the reasons why chronic use of sedative medication is strongly discouraged. Finally, a common side-effect of prescription sleep medication is daytime drowsiness. This has resulted in napping during the day, which in turn affects nocturnal sleep. Reduced daytime waking hours have been found to be negatively associated with PA participation [37]. Furthermore, Landi and colleagues [38] found that individuals who engaged in PA consumed less sleep medication than their inactive counterparts. Putting it all together, PA participation is negatively associated with dependency on sleep medication. Reduced medication use is positively associated with increase in daytime waking. When one experiences an increase in daytime waking, this individual may be more inclined to increase PA participation, which is associated with decreased dependency on sleep medication as stated. Therefore, PA may actually trigger a snowball effect to improve the quality-of-life of PWD by improving their sleep.

Our second study hypothesis stated that PWD who engaged in moderate PA had better quality sleep than their non-active counterparts. We found that there were indeed more active PWD in the good and moderate sleep group than the inactive PWD and the difference was statistically significant. Unfortunately, research examining PA on sleep quality was also scarce. This was not totally unexpected given the complexity associated with measuring such an intricate entity. Nevertheless, multiple studies had found that sleep quality, such as frequency and duration of nighttime awakenings, were significantly improved by being physically active [16,17,32]. Our study utilized six aspects of sleep quality that included restlessness, sleep onset latency, frequency and duration of nighttime awakenings, daytime drowsiness, and daytime napping. Together, we found that active PWD were more likely to experience better sleep quality and to achieve significantly higher sleep quality scores.

OTC and prescription sedative medication usage has been heavily scrutinized in the literature. In our study, we found no significant difference between the sleep quality of PWD who used OTC and/or prescription sleep medication and those who did not. This finding is in agreement with existing research [39]. As the potential side effects of sedative medication and OTC sleep medication, such as amnesia, disorientation, daytime fatigue, and excessive napping, can be devastating, pharmacological sleep aids must be used judiciously [12]. Taken together, this study supports the notion that non-pharmacological interventions should be considered as a first-line treatment against poor sleep quality.

## 5. Conclusions

Overall, research on sleep in the dementia population is sparse. Among the limited research, most focused on individuals who are already institutionalized. Sleep disturbance is among the most important reasons that PWD are admitted to an institution. As the majority of PWD would prefer to stay at home for as long as they can [40], finding effective interventions to improve sleep could potentially improve the quality-of-life for the individual and their caregiver, and possibly delay institutionalization which can put a heavy toll on the family, both emotionally and financially. Likely due to a small sample size, our study yielded several non-significant results. The nature of our study that utilized a convenient sample also likely added to the sampling error. Still, our positive findings on PA and sleep warrant future studies to determine if a definite relationship exists, and, if so, what modality and level of PA shows the greatest potential in ensuring that this benefit occurs. It is important to note that this is a correlational study and no causality should be implied. Nonetheless, the association identified provides a foundation for future studies that can explore causal mechanisms.

## Figures and Tables

**Table 1:** Characteristics of PWD by activity level: active (≥1.5 kkd) or inactive (<1.5 kkd).

Demographic Characteristics	Inactive PWD (*n* = 18)	Active PWD (*n* = 22)	*p*-Value
Age in years (mean ± SD)	78.2 ± 6.9	78.9 ± 7.0	0.77
Time since diagnosis in months (mean ± SD)	49.3 ± 22.9	46.1 ± 32.7	0.73
Gender, n (%)			
Males	8 (44.4)	15 (68.2)	0.13
Females	10 (55.6)	7 (31.8)	
Type of dementia, n (%)			
Alzheimer’s	8 (47.1)	9 (40.9)	0.84
Vascular	2 (11.8)	4 (18.2)	
Other	7 (41.2)	9 (40.9)	
Marital status, n (%)			
Married/common-law	14 (77.8)	18 (85.7)	0.52
Single	4 (22.2)	3 (14.3)	
Education level			
Secondary and below	14 (77.8)	12 (57.1)	0.17
Any post-secondary	4 (22.2)	9 (42.9)	
Home care services, n (%)			
None	9 (50.0)	13 (59.1)	0.57
One or more	9 (50.0)	9 (40.9)	
Use of drugs for dementia, n (%)			
None	2 (11.8)	5 (23.8)	0.34
One or more	15 (88.2)	16 (76.2)	
OTC sleep medication use, n (%)			
None	16 (88.9)	21 (95.5)	0.43
One or more	2 (11.1)	1 (4.6)	
*Rx* sleep medication usage, n (%)			
None	12 (66.7)	19 (86.4)	0.14
One or more	6 (33.3)	3 (13.6)	

PWD: persons with dementia; SD: standard deviation; OTC: over the counter; Rx: prescription.

**Table 2:** Frequency and duration of physical activities performed by PWD.

Types of Physical Activity	Frequency of Activity in Days Per Week Mean ± SD		Duration of Activity in Hours Per Week Mean ± SD	
	Inactive PWD	Active PWD	Inactive PWD	Active PWD
Walking	1.94 ± 3.04	5.41 ± 2.58	0.07 ± 0.12	0.96 ± 1.0
Exercise classes	0.17 ± 0.38	0.77 ± 1.54	0.13 ± 0.30	0.30 ± 0.53
Gardening	0 ± 0	0.77 ± 1.67	0 ± 0	0.41 ± 1.13
Chair exercises	0.94 ± 2.3	0.14 ± 0.64	0.03 ± 0.08	0.02 ± 0.11
Swimming	0.28 ± 1.18	0.32 ± 1.13	0.03 ± 0.08	0.05 ± 0.21
Weight training	*	0.41 ± 1.33	*	0.07 ± 0.23
Golfing	*	0.09 ± 0.43	*	0.18 ± 0.85
Skiing	*	0.05 ± 0.21	*	0.05 ± 0.21
Home exercise	0.06 ± 0.24	*	0.01 ± 0.04	*
Tennis	*	0.05 ± .213	*	0.01 ± 0.43
Cycling	*	0.14 ± 0.64	*	0.01 ± 0.05

* no participation; SD: standard deviation.

**Table 3:** Characteristics of PWD by sleep quantity.

Variables for Comparison	Inappropriate Quantity (*n* = 19)	Appropriate Quantity (*n* = 21)	*p*-Value
Activity level, n (%)			
Active	3 (15.8)	19 (90.5)	0.000
Inactive	16 (84.2)	2 (9.5)	
Age in years (mean ± SD)	78.21 ± 7.57	78.9 ± 6.26	0.75
Time since diagnosis in months (mean ± SD)	50.47 ± 28.99	44.86 ± 28.31	0.54
Gender, n (%)			
Males	10 (52.6)	13 (61.9)	0.55
Females	9 (47.4)	8 (38.1)	
Type of dementia, n (%)			
Alzheimer’s	8 (42.1)	9 (45.0)	0.98
Vascular	3 (15.8)	3 (15.0)	
Other	8 (42.1)	8 (40.0)	
Marital status, n (%)			
Married/common-law	16 (84.2)	16 (80.0)	0.73
Single	3 (15.8)	4 (20.0)	
Education level, n (%)			
Secondary and below	14 (73.6)	12 (60.0)	0.37
Any post-secondary	5 (26.3)	8 (40.0)	
Home care services, n (%)			
None	9 (47.4)	13 (61.9)	0.36
One or more	10 (52.6)	8 (38.1)	
Use of drugs for dementia, n (%)			
None	1 (5.6)	6 (30.0)	0.05
One or more	17 (94.4)	14 (70.0)	
OTC sleep medication use, n (%)			
None	17 (89.5)	20 (95.2)	0.49
One or more	2 (10.5)	1 (4.8)	
Rx sleep medication use, n (%)			
None	12 (63.2)	19 (90.5)	0.04
One or more	7 (36.8)	2 (9.5)	

SD: standard deviation; OTC: over the counter; Rx: prescription.

**Table 4:** Characteristics of PWD by sleep quality.

Variables for Comparison	Poor Sleep Quality (*n* = 11)	Moderate Sleep Quality (*n* = 18)	Good Sleep Quality (*n* = 11)	*p*-Value
Activity level, n (%)				
Active	2 (18.2)	10 (55.6)	10 (90.9)	0.003
Inactive	9 (81.8)	8 (44.4)	1 (9.1)	
Age in years (mean ± SD)	77.1 ± 5.7	79.6 ± 8.1	78.4 ± 5.8	0.78
Months since diagnosis (mean ± SD)	43.0 ± 25.2	55.7 ± 34.2	38.6 ± 25.2	0.48
Gender, n (%)				
Males	7 (63.6)	10 (55.6)	6 (54.5)	0.89
Females	4 (36.4)	8 (44.4)	5 (45.5)	
Type of dementia, n (%)				
Alzheimer’s	4 (36.4)	10 (58.8)	3 (27.3)	0.36
Vascular	1 (15.4)	3 (17.6)	2 (18.2)	
Other	6 (41)	4 (23.5)	6 (54.5)	
Marital status, n (%)				
Married/common-law	11 (100)	12 (66.7)	9 (90)	0.06
Single	0 (0.0)	6 (33.3)	1 (10)	
Education level, n (%)				
Secondary and below	6 (54.5)	13 (76.5)	7 (63.6)	0.47
Any post-secondary	5 (45.5)	4 (23.5)	4 (36.4)	
Home care services, n (%)				
None	5 (45.5)	9 (50)	8 (72.7)	0.37
One or more	6 (54.5)	9 (50)	3 (27.3)	
Use of drugs for dementia, n (%)				
None	2 (20)	3 (16.7)	2 (20)	0.97
One or more	8 (80)	15 (83.3)	8 (10)	
OTC sleep medication use, n (%)				
None	10 (90.9)	17 (94.9)	10 (90.9)	0.92
One or more	1 (9.1)	1 (5.6)	1 (9.1)	
Rx sleep medication usage, n (%)				
None	7 (63.6)	13 (72.2)	11 (100)	0.10
One or more	4 (36.4)	5 (27.8)	0 (0.0)	

SD: standard deviation; OTC: over the counter; Rx: prescription.
